# Dietary Magnesium Intake Is Inversely Associated With Ulcerative Colitis: A Case–Control Study

**DOI:** 10.1093/crocol/otae009

**Published:** 2024-02-21

**Authors:** Omid Sadeghi, Zeinab Khademi, Parvane Saneei, Ammar Hassanzadeh-Keshteli, Hamed Daghaghzadeh, Hamid Tavakkoli, Peyman Adibi, Ahmad Esmaillzadeh

**Affiliations:** Nutrition and Food Security Research Center, Department of Community Nutrition, Student Research Committee, School of Nutrition and Food Science, Isfahan University of Medical Sciences, Isfahan, Iran; Department of Community Nutrition, School of Nutritional Sciences and Dietetics, Tehran University of Medical Sciences, Tehran, Iran; Food Security Research Center, Isfahan University of Medical Sciences, Isfahan, Iran; Department of Community Nutrition, School of Nutrition and Food Science, Isfahan University of Medical Sciences, Isfahan, Iran; Department of Medicine, University of Alberta, Edmonton, Canada; Integrative Functional Gastroenterology Research Center, Isfahan University of Medical Sciences, Isfahan, Iran; Integrative Functional Gastroenterology Research Center, Isfahan University of Medical Sciences, Isfahan, Iran; Integrative Functional Gastroenterology Research Center, Isfahan University of Medical Sciences, Isfahan, Iran; Integrative Functional Gastroenterology Research Center, Isfahan University of Medical Sciences, Isfahan, Iran; Department of Community Nutrition, School of Nutritional Sciences and Dietetics, Tehran University of Medical Sciences, Tehran, Iran; Department of Community Nutrition, School of Nutrition and Food Science, Isfahan University of Medical Sciences, Isfahan, Iran; Obesity and Eating Habits Research Center, Endocrinology and Metabolism Molecular–Cellular Sciences Institute, Tehran University of Medical Sciences, Tehran, Iran

**Keywords:** magnesium, ulcerative colitis, inflammatory bowel disease, nutrition epidemiology, case–control

## Abstract

**Background:**

Ulcerative colitis (UC) causes long-lasting inflammation and ulcers in the gut. Limited observational data are available linking dietary magnesium intake and UC. In the present study, we aimed to investigate the association between dietary magnesium intake and UC in adults.

**Methods:**

The current population-based case–control study was performed on 109 UC patients and 218 age (±2 years) and sex-matched controls. The diagnosis of UC was made according to the standard criteria by a gastroenterology specialist. Dietary intakes were assessed using a validated self-administrated 106-item dish-based Food Frequency Questionnaire (FFQ). We also used a pretested questionnaire to collect data on potential confounders.

**Results:**

Individuals in the top tertile of magnesium intake were less likely to have UC compared with those in the bottom tertile. A significant inverse relationship was found between dietary magnesium intake and UC (odds ratio [OR]: 0.32, 95% confidence interval [CI]: 0.18–0.59) in the crude model. This relationship was also observed when we took several potential confounding into account (OR: 0.30, 95% CI: 0.14–0.68).

**Conclusions:**

Adherence to a magnesium-rich diet may have a role in preventing UC. However, further studies are needed to confirm our findings.

## Introduction

Magnesium is an essential mineral that plays an important role in multiple physiologic functions.^1,2^ Whole grains, vegetables, nuts, and legumes are among the main dietary sources of this mineral.^3,4^ Given the observed nutrition transition around the world, consumption of these food groups has considerably decreased, which might result in a high prevalence of magnesium deficiency around the world.^5^ Magnesium deficiency has been found to be associated with an increased risk of several chronic diseases such as cardiovascular diseases (CVDs), diabetes, and depression.^6–9^ However, limited data are available on the association between dietary magnesium intake and inflammatory-based gastrointestinal disorders including ulcerative colitis (UC). There are possible mechanisms which support magnesium involvement in UC pathogenesis including anti-inflammatory activities of magnesium,^6^ its critical role in immune response,^10^ and the possible effect of this nutrient in the modulation of gut microbiome.^11,12^ Previous studies on the link between diet and UC have found consumption of vegetables, fruits, and legumes inversely and those of sugar-sweetened beverages positively associated with UC.^13–15^ The protective association of vegetables and legumes might be attributed to their high magnesium content. With regard to dietary magnesium, we are aware of only 2 studies that investigated the association with UC.^16,17^ In a case–control study, patients with UC had higher dietary magnesium intake than healthy individuals.^16^ However, Kobayashi et al.^17^ did not find any significant association between patients with UC and healthy controls in terms of dietary magnesium intake. Both earlier studies have only compared mean dietary magnesium intake between patients with UC and controls. They also did not consider several confounding variables to find an independent association. Therefore, in the current study, we aimed to find the association between dietary magnesium intake and odds of UC in a sample of Iranian adults, considering the above-mentioned points.

## Materials and Methods

### Study Design and Participants

This population-based case–control study was carried out among a group of adult people in Isfahan, Iran. Cases with UC were adults (aged >18 years) with a confirmed diagnosis of UC by a gastroenterologist. UC patients were registered in the inflammatory bowel disease (IBD) registry database of Isfahan. All registered patients in the database (*n* = 140) were invited to participate in an educational class on lifestyle modification. In that class, information about our study design and aims was explained, and patients were requested to participate in this study. Out of these 140 patients, 119 people agreed to take part in the current study. For each patient with UC, 2 apparently healthy controls were randomly selected after matching for age (±2 years) and sex from our previous large population-based study, namely SEPAHAN, on 8000 apparently healthy individuals.^18^ Before the selection of controls, we first excluded all individuals with gastrointestinal disorders (including Crohn’s disease [CD], UC, irritable bowel syndrome, functional dyspepsia, and gastroesophageal reflux disorder) from the whole dataset. Overall, 327 participants, including 109 UC patients and 218 healthy individuals, remained for the final analysis.

### Assessment of Dietary Intakes

The usual dietary intakes of all study participants, including controls, during the past year, were assessed using a validated self-administrated 106-item dish-based Food Frequency Questionnaire (FFQ). Detailed information about the questionnaire, as well as its design and validity, has been published elsewhere.^19^ In brief, the questionnaire consisted of 106 food items with commonly used portion sizes among Iranians. The questionnaire consisted of 5 categories, including (1) mixed dishes, including cooked or canned foods (*n* = 29); (2) potatoes and grain-based foods (*n* = 10); (3) dairy products, including milk and other dairy foods, for example, butter and cream (*n* = 9); (4) fruit and vegetables (*n* = 22); and (5) miscellaneous foods and beverages, such as sweets, fast foods, prepared meals, nuts, desserts, and beverages (*n* = 36). Participants were able to report their dietary intakes through 9 multiple-choice frequency categories, including “never or less than once a month” to “12 or more times per day.” Daily intakes of each food item were then converted to grams per day using household measures.^20^ We used the Nutritionist IV to estimate total daily energy and nutrient intakes, including mean intake of magnesium. Nutritionist IV which has been modified for Iranian foods is originally based on the United States Department of Agriculture food composition table.

The validity and reliability of the FFQ were examined in a validation study on a subgroup of 200 randomly selected participants of SEPAHAN project.^19^ In that study, the FFQ was completed by all participants at baseline and 6 months later. During these 6 months, participants provided 3 detailed dietary records that were used as the gold standard. Based on the findings from this study, the FFQ could provide reasonably valid and reliable measures of long-term dietary intakes in the Iranian population; for instance, dietary carbohydrate intake obtained from the FFQ was correlated with the one obtained from the average of 3 dietary records (*r* = 0.81). Earlier studies have indicated that data on food groups’ intakes, as well as nutrient intakes, from this questionnaire provide reasonably valid data on dietary intakes.^21,22^

### Assessment of UC

The diagnosis of UC was made according to the international criteria^23^ including pathology and colonoscopy data by a gastroenterology specialist. In addition, the medical records of all cases were reviewed to confirm the diagnosis.

### Assessment of Other Variables

Information about participants’ age, sex (male/female), marital status (married/single/divorced), education (under-university/university educated), smoking (nonsmoker/ex-smoker/current smoker), house ownership (owner/nonowner), family size (≥4/<4 persons), and existence of diabetes (yes/no) and hypertension (yes/no) was gathered using pretested questionnaires. Assessment of anthropometric measures, including weight and height, was done using a validated self-administered questionnaire. Then, participants’ body mass index (BMI) was calculated as weight in kilograms divided by height in meters squared. According to a previous investigation, self-administered questionnaire of anthropometric measures provides valid information compared with actual measured values.^24^

### Statistical Analysis

We first obtained the tertile-cutoff points of dietary magnesium intake based on the dietary intakes of controls (T1: <272 mg/day, T2: between 272 and 336 mg/day, and T3: >336 mg/day), and then we categorized cases and controls based on these cutoff points. Independent sample *t*-test and one-way analysis of variance (ANOVA) were used to determine differences in continuous variables between cases and controls as well as across tertiles of dietary magnesium intake. To examine the distribution of participants in terms of categorical variables, we applied the Chi-square test. Binary logistic regression with adjustment for covariates was used to obtain the odds ratios (ORs) (95% confidence intervals [CIs]) for UC across the tertiles of dietary magnesium intake. In the first model, we adjusted for age, sex, and energy intake. Additional adjustment was made for education, marital status, smoking, house possession, family size, diabetes, and hypertension in the second model. Then, we did further control for BMI to obtain an obesity-independent association. In these analyses, participants in the first tertile of dietary magnesium intake were considered the reference group. To obtain the trend of ORs across the increasing tertiles of magnesium intake, we considered tertiles as an ordinal variable. All statistical analyses were conducted using the SPSS software (version 19.0; SPSS Inc., Chicago, IL). *P*-values less than .05 were considered significant.

## Results

In the present study, median magnesium intake across increasing tertiles was 205, 311, and 426, respectively. General characteristics of cases and controls across tertiles of dietary magnesium intake are presented in Table 1. Patients with UC were less likely to be university graduates and more likely to be from a large family size compared to controls. No other significant differences were seen between cases and controls. No overall significant difference was seen in terms of these variables across tertiles of dietary magnesium intake.

**Table 1.** General characteristics of cases and controls across tertiles of dietary magnesium intake.

	UC			Magnesium intake			
Variables	No (*n* = 218)	Yes (*n* = 109)	*P*-value^a^	T1 (*n* = 135)	T2 (*n* = 98)	T3 (*n* = 94)	*P*-value^b^
Age (years)	39.5 ± 10.0	41.5 ± 11.8	.12	40.16 ± 11.23	39.87 ± 9.13	40.48 ± 11.36	.92
Male (%)	47.7	48.1	.94	50.7	46.9	44.7	.65
BMI (kg/m^2^)	25.3 ± 3.8	25.1 ± 3.4	.72	25.27 ± 3.84	25.56 ± 4.00	25.03 ± 3.29	.63
Married (%)	80.3	77.9	.61	80.3	76.3	81.7	.62
University graduated (%)	54.6	38.0	.005	42.5	50.0	57.4	.08
Family size (≥4) (%)	12.4	56.5	<.001	32.1	23.5	23.4	.22
House possession (owner) (%)	71.1	80.0	.09	75.8	75.6	71.1	.71
Current smoker (%)	4.1	6.0	.12	8.9	12.2	15.7	.33
Hypertension (%)	6.0	8.3	.46	6.7	4.1	9.6	.31
Diabetes (%)	2.3	3.7	.48	3.7	1.0	2.8	.44

Data are presented as mean ± SD or percent.

Abbreviations: BMI, body mass index; SD, standard deviation.

^a^Obtained from the independent sample *t*-test or Chi-square test, where appropriate.

^b^Obtained from the analysis of variance (ANOVA).

Dietary intakes of selected food groups and nutrients across tertiles of magnesium intake are indicated in Table 2. Individuals in the highest tertile of magnesium intake had greater intakes of whole grains, dairy, fruits, vegetables, nuts, energy, fat, protein, carbohydrates, dietary fiber, vitamins E, D, and B6, iron, and caffeine compared with those in the lowest tertile.

**Table 2.** Dietary intakes of study participants across tertiles of dietary magnesium intake.

	T1	T2	T3	*P*-value^a^
Energy (kcal/day)	1895.6 ± 63.2	2588.3 ± 65.5	3554.1 ± 124.9	<.001
Food groups				
Whole grains (g/day)	59.7 ± 3.7	77.0 ± 4.7	153.4 ± 14.6	<.001
Dairy (g/day)	217.4 ± 14.6	314.0 ± 20.0	457.2 ± 37.6	<.001
Fruits (g/day)	307.3 ± 20.8	375.3 ± 31.0	600.1 ± 53.0	<.001
Vegetables (g/day)	142.4 ± 6.2	221.5 ± 12.8	292.1 ± 15.2	<.001
Nuts (g/day)	5.4 ± 0.6	8.3 ± 0.9	13.5 ± 2.3	<.001
Nutrients				
Protein (g/day)	67.9 ± 2.2	95.3 ± 2.4	131.5 ± 4.9	<.001
Carbohydrates (g/day)	227.3 ± 9.5	315.7 ± 11.3	451.2 ± 17.3	<.001
Fat (g/day)	82.5 ± 3.0	109.1 ± 3.3	142.6 ± 6.3	<.001
Total fiber (g/day)	14.0 ± 0.3	22.1 ± 0.6	32.7 ± 0.9	<.001
Vitamin E (mg/day)	13.4 ± 0.4	21.3 ± 0.6	26.7 ± 0.9	<.001
Vitamin D (mcg/day)	0.6 ± 0.04	1.1 ± 0.1	1.2 ± 0.09	<.001
Vitamin B6 (mg/day)	1.32 ± 0.02	2.03 ± 0.04	2.8 ± 0.07	<.001
Iron (mg/day)	12.2 ± 0.4	18.3 ± 0.5	26.0 ± 0.9	<.001
Caffeine (mg/day)	70.8 ± 5.2	89.7 ± 6.8	101.3 ± 10.1	.009

Data are presented as mean ± SD.

Abbreviation: SD, standard deviation.

^a^Obtained from the analysis of variance (ANOVA).

Multivariable-adjusted ORs and 95% CIs for UC across tertiles of magnesium intake are presented in Table 3. A significant inverse association was seen between dietary magnesium intake and UC (OR: 0.32, 95% CI: 0.18–0.59). This inverse relationship was also observed after taking age, sex, and energy intake into account (OR: 0.30, 95% CI: 0.16–0.56). Further adjustment for marital status, education, family size, house possession, hypertension, diabetes, and smoking did not change this association (OR: 0.33, 95% CI: 0.15–0.72). Further adjustment for BMI also resulted in a significant inverse association (OR: 0.30, 95% CI: 0.14–0.68).

**Table 3.** Multivariable-adjusted odds ratios for ulcerative colitis across tertiles of dietary magnesium intake.

	Tertiles of magnesium intake			
	T1	T2	T3	*P*-trend^a^
Median of magnesium intake (mg/day)	205	311	426	
Crude	1.00	0.39 (0.22–0.68)	0.32 (0.18–0.59)	.001
Model 1	1.00	0.36 (0.20–0.66)	0.30 (0.16–0.56)	<.001
Model 2	1.00	0.44 (0.21–0.93)	0.33 (0.15–0.72)	.003
Model 3	1.00	0.40 (0.19–0.86)	0.30 (0.14–0.68)	.002

Data are presented as OR (95% CI).

Abbreviations: BMI, body mass index; CI, confidence interval; OR, odds ratio.

Model 1: adjusted for age, sex, and energy intake.

Model 2: further adjustments for marital status, education, family size, house possession, hypertension, diabetes, and smoking.

Model 3: additionally, adjusted for BMI.

^a^Obtained from binary logistic regression.

## Discussion

In the present study, we found that individuals with high intake of dietary magnesium were less likely to have UC compared with those having a low intake. This inverse association was also seen after controlling for potential confounding variables. This study is the first to determine an independent association between dietary magnesium intake and UC.

UC is a disabling disorder that is among the most prevalent gastrointestinal diseases in the world.^25^ UC affects several aspects of life in those who suffer from it. High prevalence of psychological disorders and malnutrition has been reported in these patients. UC also considerably reduces the quality of life in the patients.^26,27^ Evidence has shown that dietary factors have an important role in the incidence and management of UC.^28,29^ Previous studies have mainly focused on the contribution of diet to the management of UC symptoms,^30,31^ whereas little attention has been paid to the role of dietary factors in the prevention or incidence of this disorder.^32^ In the current study, we found that dietary magnesium intake was associated with reduced odds of UC. Although few studies are available on the association between dietary magnesium intake and UC, our findings are comparable with the studies on magnesium-rich foods. For instance, in a study on 230 IBD patients and 12 462 healthy individuals, intakes of dietary sources of magnesium such as fruit and beans/legumes were inversely associated with UC.^33^ Unlike our observations, in a case–control study, although UC patients had a significantly higher dietary magnesium intake than healthy individuals, no significant association between magnesium intake and odds of UC was found.^16^ Moreover, Kobayashi et al.^17^ reported that magnesium intake had no significant association with UC. This conflict in findings might be due to the influence of disease on the food preferences of patients. UC patients might change their diet to have healthier dietary patterns, which are usually rich in magnesium. Furthermore, considering the different variables as confounders and different populations or sample sizes, there might be other reasons for the observed discrepancy.

Given the inflammatory nature of UC, magnesium may have a protective association with UC through its anti-inflammatory effects.^34,35^ It has been shown that magnesium suppresses the lipopolysaccharide (LPS)-induced activation of phospholipase A2 (PLA2) and the production of arachidonic acid (AA) metabolites such as prostaglandin E2 (PGE2), prostacyclin (PGI2), thromboxane 2 (TXB2), and leukotrienes (LTB4) in macrophages.^36,37^ Moreover, LPS-induced AA-metabolizing enzymes, including cyclooxygenase, lipoxygenase, and prostaglandin I2 synthase, were significantly inhibited by magnesium.^37^ These metabolites and enzymes are involved in inflammatory pathways. In addition, experimental studies have indicated that magnesium deficiency or adherence to a magnesium-deficient diet might induce an imbalance in gut microbiota.^38,39^ Imbalances in gut microbiota can adversely affect gut barrier integrity, increase inflammation in the gut, and consequently increase the incidence of IBD.^40,41^

The current study has several strengths. This is the first study examining the independent association between dietary magnesium intake and UC by taking several confounders into account. We used a validated FFQ to collect dietary data. However, during the interpretation of our findings, some limitations should also be noticed. The main limitation is the case–control design of our study, which prohibits us from inferring a causal link between magnesium intake and UC. Therefore, further prospective studies are needed to confirm our findings. Moreover, adherent selection and recall biases in case–control studies should also be considered. As with all epidemiological studies, misclassification of study participants due to the use of FFQ is unavoidable. Additionally, despite controlling for several confounding variables, we cannot exclude the effect of residual confounders such as history of medication intake. The possibility of changes in usual dietary intake as a result of UC patient’s condition must be considered as well. The generalizability of study findings might be weakening with this methodological choice.

In conclusion, a greater intake of dietary magnesium was associated with reduced odds of UC in Iranian adults. Further studies are needed to confirm our findings. Also, it is recommended that future studies examine the effects of a magnesium-rich diet or magnesium supplementation on the symptoms of UC.

## Data Availability

The datasets analyzed during the current study are available from the corresponding author upon reasonable request.
